# Surgical Challenges Posed by Anomalous Biliary Tree Anatomy in Choledochal Cyst Excision: A Case Report

**DOI:** 10.7759/cureus.83387

**Published:** 2025-05-03

**Authors:** Sumit Boricha, Girish Bakhshi, Amit A Thombare, Urvashi Jain, Sakshi M Jain, Avinash Gutte, Kuldeep Kapdi

**Affiliations:** 1 General Surgery, Grant Medical College (GMC) and Sir J.J. Group of Hospitals, Mumbai, IND; 2 Radiology, Grant Medical College (GMC) and Sir J.J. Group of Hospitals, Mumbai, IND

**Keywords:** biliary-enteric anastomosis, biliary tract reconstruction, biliary tract variation, choledochal cyst excision, hepato-pancreato-biliary surgery

## Abstract

A choledochal cyst (CC) usually presents with abdominal pain, jaundice, and a palpable abdominal mass. CC usually results in complications like cystolithiasis, choledocholithiasis, pancreatitis, liver cirrhosis, and intracystic stones, which are particularly more common in Type 4 CC. Here we present a rare case of Type 4a and Type 6 CC in a 21-year-old female patient who presented to us with complaints of acute onset of jaundice and pain in the abdomen. She was evaluated preoperatively with magnetic resonance cholangiopancreaticography (MRCP) and subsequently planned for excision of the cyst with hepaticojejunostomy as a definite mode of management. Intraoperative findings required a double bilioenteric anastomosis. This case emphasizes that in spite of preoperative imaging and radio-diagnosis, the intraoperative findings may differ. Hence, a surgeon should be mentally prepared to do intraoperative biliary tree exploration and modify the treatment plan accordingly.

## Introduction

Choledochal cysts (CC) are rare congenital abnormalities characterized by intra- and/or extrahepatic dilatation of biliary radicals. Choledochal cyst is an uncommon anomaly and is estimated to occur in one in 13,000 to one in two million live births. Approximately 80% of CCs are diagnosed in childhood and are more common in females [1]. These cysts present with abdominal pain, jaundice, and sometimes a palpable abdominal mass. The management of CCs involves surgical excision of the cyst along with bilioenteric anastomoses. The treatment of Type 4 CCs remains challenging as they are associated with complex anatomical variations both in the intrahepatic and extrahepatic biliary tree.

## Case presentation

**Figure 1:**
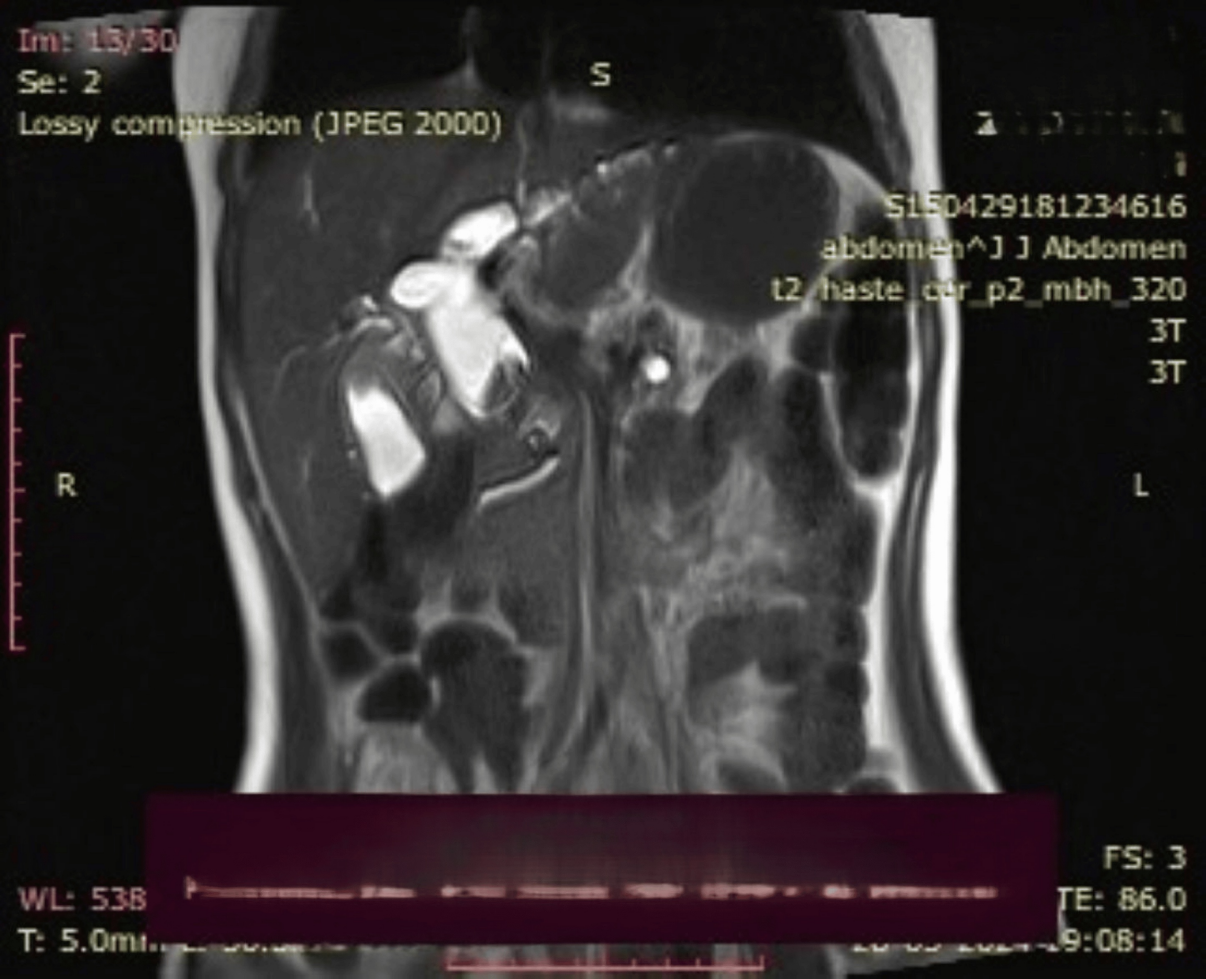
A dilated cystic duct with a dilated common hepatic duct and choledochal cyst is visualized

**Figure 2:**
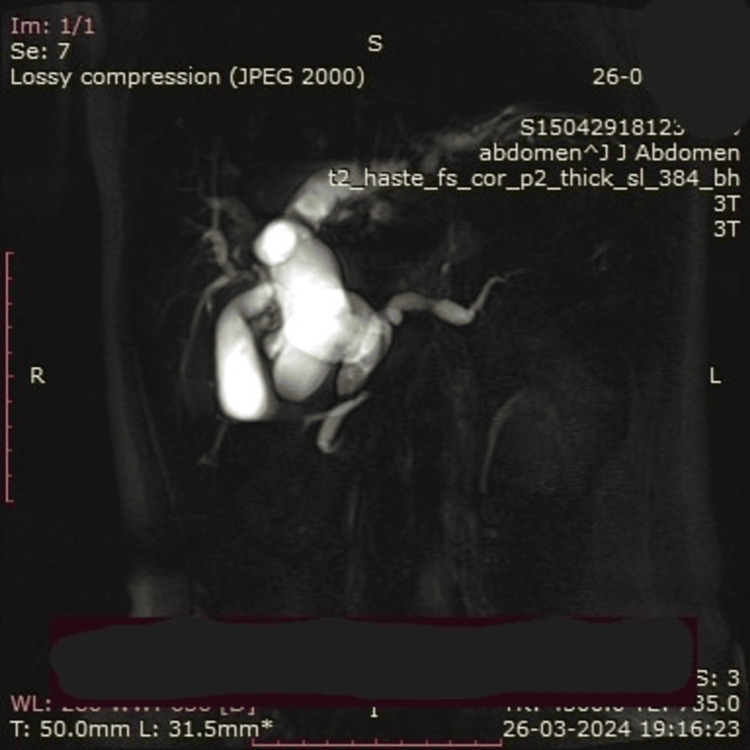
A dilated cystic duct with a choledochal cyst with faint hyperintensities in segments 6, 7, and 8

A 21-year-old female patient presented with pain in the abdomen, occasional episodes of vomiting, and jaundice for 10 days. Clinical examination revealed icterus and tenderness in the right hypochondrium. Ultrasound of the abdomen and pelvis was suggestive of the common bile duct grossly dilated along the entire length, showing a fusiform appearance in the mid-part with associated central and peripheral intrahepatic biliary radical dilatation (IHBRD). The gallbladder appeared distended and had irregular wall thickening with multiple hyperechoic foci with a dilated cystic duct (CD). The pancreatic body appeared atrophic and echogenic in the main pancreatic duct (MPD). Magnetic resonance cholangio-pancreaticography (MRCP) showed a dilated common bile duct with central and peripheral IHBRD suggestive of a CC (Type 4a) with pancreatic divisum (Figures 1, 2). On MRCP imaging, there was evidence of dilated CD and dilated common hepatic duct (CHD), suggestive of Type 4 and Type 6 CC. There was a long common channel biliary and pancreatic duct, of 2.5cm. The CD was seen opening into the CHD lower down.

Intraoperative findings

The patient was taken up for surgical intervention after workup. Intraoperatively, the CD and the CC were seen in close proximity with the portal vein. Once the complex of CC with CD was separated from the portal vein, it was transected near the lower end, just near the head of the pancreas. The lower end, going towards the ampulla, was closed in two layers using polygalactin sutures.

**Figure 3:**
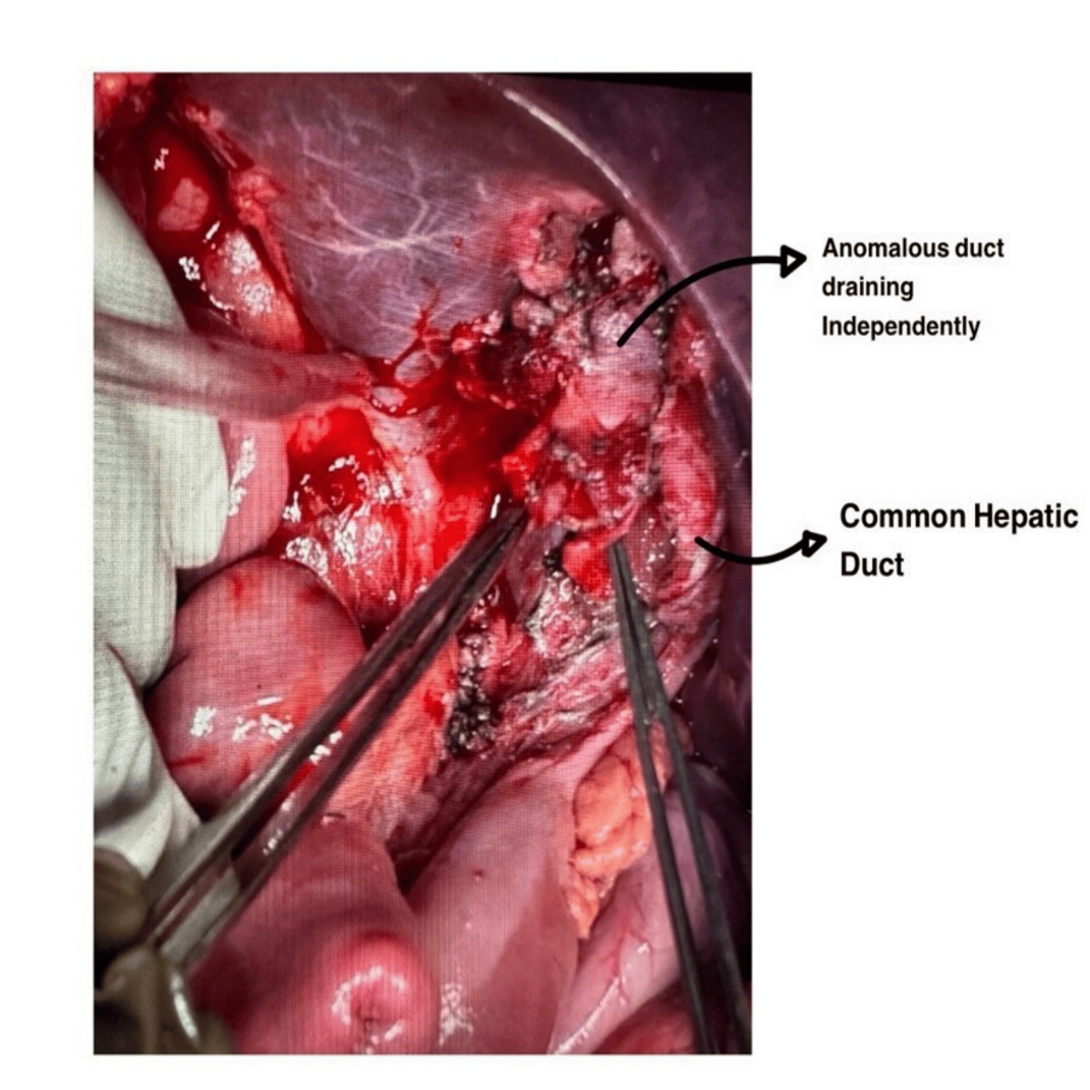
Intraoperative image after the excision of the gallbladder and the choledochal cyst; two separate ducts are seen clearly.

The CC and CD complex was traced till the porta, and the CC was excised. Following this, we were left with two duct channels. One was identified as the CHD, and the other was identified as a CD (Figure 3). A urethral dilator was passed from the identified CHD, which was seen entering the right hepatic duct and left hepatic duct of the liver. Hence, confirming the ductal opening to be a CHD. The neighboring duct, which was supposed to be the CD, was also examined with a urethral dilator. The urethral dilator was seen entering the gallbladder, but there was an anomalous ductal opening along the wall of the CD, which couldn’t be negotiated with any instruments. There was a free flow of bile seen coming from the anomalous duct even after clamping the gallbladder at the neck. It was concluded that these channels were draining separately; hence, it was decided that these structures would be included in the anastomosis. Cholecystectomy was done by ligating the CD close to the neck of the gallbladder. This was followed by a double bilioenteric anastomosis, one with CHD and the other with CD, so as to include the anomalous duct draining bile.

A subhepatic and pelvic drain was kept. The patient was started on a liquid diet on postoperative day 2 and subsequently on a full diet. The pelvic drain was removed on postoperative day 3 and subhepatic on postoperative day 5, and the patient was discharged.

**Figure 4:**
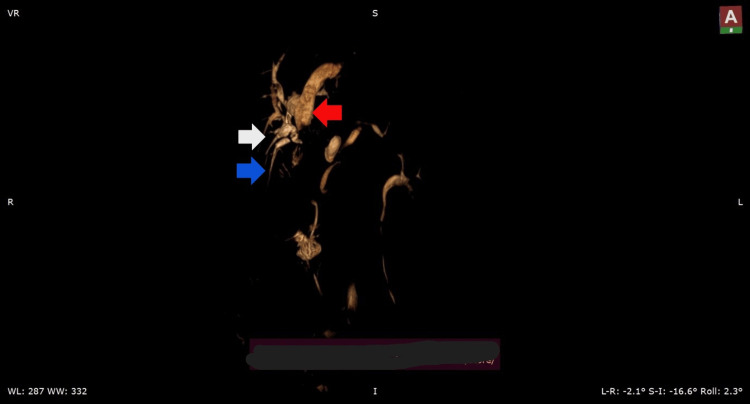
Postoperative 3D reconstructed magnetic resonance cholangiopancreatography (MRCP) images red arrow: common hepatic duct (CHD), white arrow: cystic duct (CD), blue arrow: anomalous tract seen in close proximity to the CD draining segment 6

On postoperative day 15, a repeat MRCP was done to look for the anomalous ducts, and 3D reconstructed images were obtained, which confirmed the intraoperative findings. The anomalous duct was seen draining segment 6 and opening into the CD (Figure 4).

**Figure 5:**
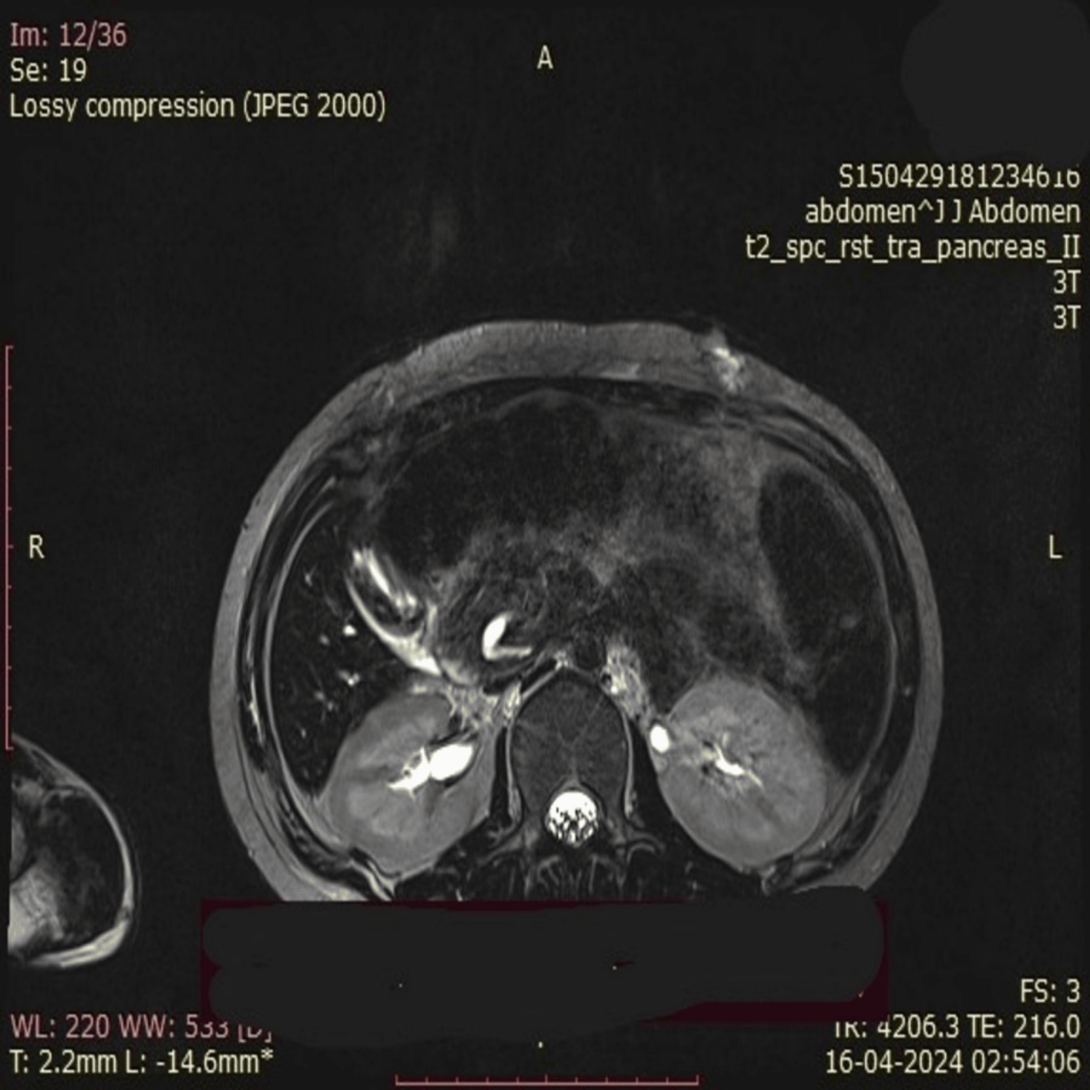
Magnetic resonance cholangiopancreatography (MRCP) image representing the anomalous tract arising from the head of the pancreas, draining into the main pancreatic duct (MPD)

Along with an aberrant biliary tree anatomy, the patient also had an aberrant pancreatic duct anatomy (Figure 4). There was one minor pancreatic duct from the head of the pancreas draining into the CBD directly and another minor duct draining from the uncinate process of the pancreas into the long common channel of the pancreatico-biliary malunion (PBMU) (Figure 5), which to our knowledge has not been reported yet in the literature [2].

## Discussion

Surgical excision of the CC with biliary tract reconstructions remains the foundation in the management of CC in most age groups. While hepaticoduodenostomy and hepaticojejunostomy both successfully restore the biliary flow, even large meta-analytical studies have been inconclusive as to which technique is better than the other when it comes to long-term survival and complications [3].

Although multiple long-term studies and recent trends indicate that a complete cyst excision with a Roux-en-Y hepaticojejunostomy is the most standard modality of management of Type 1, 4b, and certain Type 4a, which provides decent, favorable short-term and long-term outcomes [4].

The case presented here, involving a 21-year-old female patient with Type 4a and Type 6 CC, posed several challenges, particularly due to the complexity of the biliary anatomy.

Preoperative imaging, specifically MRCP, was critical in defining the anatomical landscape of the biliary tree, highlighting key features such as a dilated common bile duct and the presence of a long common pancreaticobiliary channel. But failed to delineate complex biliary tree anatomy, which was encountered intraoperatively.

Intraoperatively, the anomalous relationship between the CD, CHD, and CC was evident. The presence of an anomalous duct draining segments 5 and 6 into the CD added complexity to the existing varied anatomy in a CC case. Such aberrant anatomy can complicate the identification and dissection of the biliary structures, increasing the risk of vascular or ductal injury.

A key challenge in this case was the identification and management of multiple biliary channels. The need to perform a double bilioenteric anastomosis highlights the importance of detailed anatomical knowledge and careful intraoperative exploration. In addition to the CHD, an anomalous duct draining into the CD required separate anastomosis to ensure adequate biliary drainage and prevent postoperative complications like bile leakage.

Moreover, the patient exhibited a rare pancreatic duct anomaly, with dual minor ducts draining from the head and uncinate process of the pancreas into the common bile duct and a common channel, respectively. This unusual ductal configuration has not been widely reported and adds another layer of complexity to the case, further underscoring the variability of biliary and pancreatic anatomy in CC patients.

This case report not only showcases one-of-a-kind anatomical variations in the biliary tree and the pancreatic duct anatomy but also highlights the importance of preoperative imaging techniques, emphasizing more on the involvement of a multi-disciplinary approach in the management of these patients by discussing preoperative imaging with the radiodiagnosis department of the institute. At the same time, the healthcare professional must be ready to deal with intraoperative surgical challenges posed due to the varied anatomy of the biliary tree while making the best possible surgical choices for the patient's benefit, which was reconfirmed with a postoperative check scan.

Similarly stated in a case report, it highlights a rare presentation of biliary anatomy and suggests having a high index of suspicion while operating on a CC and preventing any inadvertent injury to the biliary tree or to the adjacent vital structures [5].

## Conclusions

This case underscores the vital importance of a surgeon’s preparedness to encounter and adapt to unanticipated anatomical variations, despite thorough preoperative imaging. While MRCP remains an invaluable tool in planning for CC excision, it may not always delineate intricate anomalies, such as aberrant ductal openings or accessory ducts. In this case, the intraoperative finding of an anomalous duct draining segment VI into the CD necessitated a strategic departure from the standard surgical plan, leading to the rare implementation of a double bilioenteric anastomosis. Furthermore, the identification of a concurrent pancreatic ductal anomaly, previously unreported to this extent, highlights the embryological and clinical complexity often masked within the spectrum of biliary cystic diseases. This reaffirms the need for not only anatomical vigilance but also a high index of suspicion during biliary surgery.

This case also emphasizes the role of postoperative imaging in confirming intraoperative findings and guiding long-term management. Multidisciplinary collaboration between surgeons and radiologists proves crucial, particularly in rare and complicated cases, to avoid missed anomalies and reduce the risk of postoperative complications. In conclusion, successful management of complex CCs relies not just on surgical expertise but also on the ability to dynamically integrate intraoperative findings with preoperative planning. Such rare cases broaden our understanding of biliary and pancreatic ductal anatomy and reinforce the imperative of individualized surgical strategies in congenital biliary disorders.
